# Acute treatment with relaxin protects the kidney against ischaemia/reperfusion injury

**DOI:** 10.1111/jcmm.12120

**Published:** 2013-09-20

**Authors:** Massimo Collino, Mara Rogazzo, Alessandro Pini, Elisa Benetti, Arianna Carolina Rosa, Fausto Chiazza, Roberto Fantozzi, Daniele Bani, Emanuela Masini

**Affiliations:** a Department of Drug Science and Technology, University of TurinTurin, Italy; b Department of Experimental and Clinical Medicine Research Unit of Histology and Embriology, University of FlorenceFlorence, Italy; c Department of NEUROFARBA Section of Pharmacology, University of FlorenceFlorence, Italy

**Keywords:** relaxin, renal ischaemia/reperfusion, acute kidney injury, inflammation

## Abstract

Although recent preclinical and clinical studies have demonstrated that recombinant human relaxin (rhRLX) may have important therapeutic potential in acute heart failure and chronic kidney diseases, the effects of acute rhRLX administration against renal ischaemia/reperfusion (I/R) injury have never been investigated. Using a rat model of 1-hr bilateral renal artery occlusion followed by 6-hr reperfusion, we investigated the effects of rhRLX (5 μg/Kg i.v.) given both at the beginning and after 3 hrs of reperfusion. Acute rhRLX administration attenuated the functional renal injury (increase in serum urea and creatinine), glomerular dysfunction (decrease in creatinine clearance) and tubular dysfunction (increase in urinary excretion of *N*-acetyl-β-glucosaminidase) evoked by renal I/R. These beneficial effects were accompanied by a significant reduction in local lipid peroxidation, free radical-induced DNA damage and increase in the expression/activity of the endogenous antioxidant enzymes MnSOD and CuZnSOD superoxide dismutases (SOD). Furthermore, rhRLX administration attenuated the increase in leucocyte activation, as suggested by inhibition of myeloperoxidase activity, intercellular-adhesion-molecule-1 expression, interleukin (IL)-1β, IL-18 and tumour necrosis factor-α production as well as increase in IL-10 production. Interestingly, the reduced oxidative stress status and neutrophil activation here reported were associated with rhRLX-induced activation of endothelial nitric oxide synthase and up-regulation of inducible nitric oxide synthase, possibly secondary to activation of Akt and the extracellular signal-regulated protein kinase (ERK) 1/2, respectively. Thus, we report herein that rhRLX protects the kidney against I/R injury by a mechanism that involves changes in nitric oxide signalling pathway.

## Introduction

Since its discovery in 1926, relaxin (RLX) has long been regarded as a peptide hormone of ovarian origin involved in the peri-partum widening of the pubic symphysis. Subsequently, growing evidence has suggested that RLX may exert a broad range of biological effects on many organs and apparatuses, especially the cardiovascular system 1. In animal models, RLX has been reported to increase blood perfusion and reduce myocardial, cerebral, intestinal and pulmonary ischaemic damage 2–6. In humans, clinical trials studying intravenous recombinant human RLX (rhRLX) as a treatment for patients hospitalized with acute heart failure have recently shown significant improvement in clinical outcome without any major adverse events 7–8. The effects of rhRLX treatment on placental blood flow, maternal blood pressure and renal function are also being evaluated in women with preeclampsia, a condition of new-onset hypertension and proteinuria during pregnancy 9–10.

The specific mechanisms by which RLX mediates its cardiovascular effects have still to be fully understood, but are key to identifying novel targets that may be used to enhance its therapeutic potential. Relaxin is a member of the relaxin peptide family, which, in humans, encompasses H1, H2 and H3 RLXs as well as insulin like peptides (INSL) 3, 5 and 6. The main circulating isoform is H2, equivalent to other species' relaxin-1, commonly referred to as RLX. Relaxin family peptides target specific G-protein-coupled receptors, defined relaxin family peptide receptors (RXFP), of which RXFP1 is currently considered the main and specific H2 RLX receptor 11. Recently, both RLX peptide and its receptor RXFP1 have been identified in the renal medulla and cortex, thus suggesting that the kidney is both a potential source and a target organ for RLX activity 12. Exogenous RLX administration has been reported to reduce the progression of diseases in several experimental models of renal fibrosis and the absence of endogenous RLX may contribute to the development of spontaneous fibrosis 13. In addition to its well-documented antifibrotic actions, RLX has been shown to increase renal plasma flow and glomerular filtration rate, attenuate the renal circulatory response to angiotensin II and reduce plasma osmolality 14. Although various effects of RLX in renal diseases have been discovered, the potential role of RLX in renal ischaemia/reperfusion injury (I/R), one of the most common causes of acute kidney injury (AKI), has not yet been investigated. Acute kidney injury is a major kidney disease associated with high mortality and morbidity and several large epidemiological studies have linked AKI with the later development of chronic and end-stage kidney diseases 15. Unfortunately, pharmacological interventions are limited and there is currently no successful therapy, except for supportive care. Thus, this study was designed (*i*) to investigate the effects of rhRLX-2 on renal dysfunction and injury evoked by I/R in the rat and (*ii*) to better elucidate the signalling mechanism(s) by which RLX exerts its effects on the kidney.

## Materials and methods

### Animals and surgery

Male Wistar rats (Harlan-Italy; Udine, Italy) were fed a Piccioni pellet diet (n.48, Gessate Milanese, Italy) and water *ad libitum*. Animal care was in compliance with Italian regulations on the protection of animals used for experimental and other scientific purposes (D.M. 116/92). The experimental protocol, approved by the Turin University Ethics Committee, was employed in multiple previous reports from our laboratory. The renal I/R protocol here described has been approved by the Turin University Ethics Committee and it was employed in multiple previous reports from our laboratory, resulting in significant reproducible and severe (but not fatal) renal dysfunction and injury, against which different interventions have shown beneficial effects 16,17. Briefly, the rats were anaesthetized through i.p. injection (30 mg/kg) of Zoletil® (15 mg/kg tiletamine + 15 mg/kg zolazepam; Zoletil 100®, 100 mg/ml, Laboratoires Virbac, Carros Cedex, France). The anaesthetized rats were placed onto a thermostatically controlled heating pad, a rectal temperature probe was inserted and body temperature was monitored and maintained at 37°C. A midline laparotomy was performed and the bladder was cannulated for the collection of urine. The kidneys were located and the renal pedicles, containing the renal artery, vein, and nerves, were carefully isolated. The rats were subjected to bilateral renal occlusion for 60 min. using non-traumatic artery clamps (Dieffenbach Bulldog Clamps, Harvard Apparatus Ltd., Kent, United Kingdom) to clamp the renal pedicles, followed by reperfusion for 6 hrs. Sham-operated rats underwent identical surgical procedures to those undergoing I/R except that artery clamps were not applied. At the end of the reperfusion, the anaesthetized rats were killed by decapitation after aortic exsanguination. The kidneys were isolated, weighed, rapidly freeze-clamped with liquid nitrogen and stored at −80°C until needed.

### Drugs and treatments

Recombinant human H2 RLX was dissolved in PBS (PBS) and administered at the dose of 5 μg/kg (i.v) at the beginning of reperfusion and again after 3 hrs of reperfusion. Serum concentration-time profile after iv bolus administration of rhRLX to rats has been described by three exponential terms, with *t*_1/2α_, *t*_1/2β_, and *t*_1/2γ_ in the range 1–3, 15–17, 60–70 min., respectively 19. In humans, the half-life of RLX has been assessed to be about 5–15 min. 20. Besides, RLX plasma levels have been reported to be above 40 pg/ml when measured at 18 hrs after a single subcutaneous injection of 2 μg RLX in mice 21. The dose of rhRLX used was based on what we have previously shown to reduce infarct size in an *in vivo* model of acute myocardial infarction 3.

Animals were randomly assigned to the following experimental groups:

1. Sham: rats were treated with the vehicle (PBS, *n* = 8) and subjected to the surgical procedure alone, without causing ischaemia;
2. Sham + RLX: rats were treated with rhRLX (5 μg/kg i.v.) prior to the sham operation (*n* = 8);
3. IR: rats were subjected to 1 hr ischaemia followed by 6 hrs of reperfusion and treated with the vehicle (PBS), at the beginning of reperfusion and again after 3 hrs of reperfusion (*n* = 8);
4. IR + RLX: rats were subjected to 1 hr ischaemia followed by 6 hrs of reperfusion and treated with rhRLX (5 μg/kg i.v.), at the beginning of reperfusion and again after 3 hrs of reperfusion (*n* = 8).

### Measurement of biochemical parameters

At the end of the reperfusion period, 1 ml blood samples were collected and centrifuged (10,000 × g for 10 min.) to separate the serum, from which biochemical parameters were measured within 24 hrs. The volume of urine produced was determined using the urine collected during the reperfusion period. Serum and urine creatinine concentrations were measured spectrophotometrically at 490 nm by the Jaffé kinetic reaction, using commercially available kits. Renal creatinine clearance was calculated by the standard formula C = (*U* × *V*)/*P*, where *U* is the concentration in urine, *V* is urine flow rate and *P* is the plasma concentration. Serum urea and creatinine concentrations and creatinine clearance were used as indicators of impaired renal function. *N*-acetyl-β-glucosaminidase (NAG) was measured in the urine of experimental rats by a colorimetric assay (Roche Diagnostics, Mannheim, Germany) and was used as marker of tubular injury 22.

### Histopathological examination and tissue injury scoring

Histopathological analysis was carried out on whole kidney cryostat cross-sections stained with either haematoxylin-eosin or Periodic acid-Schiff (PAS) staining for glycoproteins. The used severity scoring criteria are reported in Table 1. Each animal was assigned a separate score for glomeruli, tubuli and blood vessel injury, evaluated by two independent observers (D.B. & A.P.) blinded to the experimental groups, and the values were then averaged.

**Table 1:** Histopathological scoring criteria

Grade	Glomeruli	Proximal/distal tubuli	Blood vessels
0	Normal	Normal	Normal
1	Microvacuolation	Microvacuolation	Focal dilation and blood stasis
2	Vacuolation	Vacuolation, ruffled border disappearance, cell shedding, rare casts	Diffuse dilation and blood stasis
3	Vacuolation, cell shedding, enlargement of Bowman capsule	Vacuolation, diffuse cell detachment, many casts	Diffuse, severe dilation and blood stasis, interstitial haemorrhage

### Determination of thiobarbituric acid-reactive substances

Thiobarbituric acid-reactive substances (TBARS) are end-products of cell membrane lipid peroxidation by reactive oxygen species (ROS) and are considered a reliable marker of oxidative stress-induced cell damage. Thiobarbituric acid-reactive substances were determined by measurement of the chromogen obtained from the reaction of malondialdehyde with 2-thiobarbituric acid according to Aruoma *et al*. 23.

### Determination of 8-Hydroxy-2′-deoxyGuanosine

DNA isolation from cardiac tissue homogenates was performed according to Masini *et al*. 4. Samples of DNA extract were used for 8-Hydroxy-2′-deoxyGuanosine (8-OHdG) determination with a Bioxytech enzyme immunoassay kit (Oxis, Portland, OR, USA), following the instructions provided by the manufacturer. The values are expressed as ng 8-OHdG/μg proteins.

### Measurement of MnSOD and CuZnSOD superoxide dismutase activities

Kidney samples were homogenized with 10 mM PBS, pH 7.4, sonicated on ice (three times, 20 sec.) and centrifuged at 100 × g for 10 min. Superoxide dismutases (SOD) activity was measured in the supernatants as described by Nishida *et al*. 24, with minor modifications. The assay is based on the inhibition of nitroblue tetrazolium conversion by SOD into a blue tetrazolium salt, mediated by superoxide radicals, which are generated by xanthine oxidase. The amount required to inhibit the rate of reduction of nitro blue tetrazolium by 50% was defined as one unit of enzyme activity. Total SOD activity was determined by monitoring the rate of reduction of nitroblue tetrazolium. Activity of MnSOD was measured in the presence of 5 mM sodium cyanide and activity of CuZnSOD was calculated by subtracting MnSOD activity from total SOD activity.

### Myeloperoxidase activity

Myeloperoxidase activity, used as an indicator of leucocyte accumulation into the kidney, was determined as previously described 17. Briefly, samples were homogenized and centrifuged for 30 min. at 13,000 × g at 4°C. An aliquot of the supernatant was then allowed to react with a solution of 1.6 mM tetramethylbenzidine and 0.1 mM H_2_O_2_. The rate of change in absorbance was measured spectrophotometrically at 460 nm. Myeloperoxidase activity was defined as the quantity of enzyme degrading 1 μmol of peroxide/min. at 37°C and was expressed in milliunits/g of wet tissue.

### Determination of interleukin (IL)-1β, IL.18, tumour necrosis factor (TNF)-α and IL-10 production

Cytokines were measured with commercial ELISA kits (Cayman Chemical, Ann Arbor, MI, USA), following the protocol provided by the manufacturer.

### Western blot analysis

Western blots were carried out as previously described 25. Proteins were separated by 8% sodium dodecyl sulphate-polyacrylamide gel electrophoresis and transferred to polyvinyldenedifluoride membrane, which was then incubated with primary antibodies (goat anti-ICAM-1, mouse anti-phERK, rabbit anti-ERK, rabbit anti-iNOS, mouse anti-phAkt, rabbit anti Akt, goat anti-ph-eNOS, rabbit anti-eNOS, goat anti-CuZnSOD, rabbit anti-MnSOD). Blots were then incubated with a secondary antibody conjugated with horseradish peroxidase (dilution 1:10,000) and developed with the enhanced chemiluminescence (ECL) detection system. The immunoreactive bands were visualized by autoradiography and the density of the bands was evaluated densitometrically using Gel Pro®Analyzer 4.5, 2000 software (Media Cybernetics, Silver Spring, MD, USA). The membranes were stripped and incubated with β-actin monoclonal antibody (dilution 1:5000) and subsequently with an anti-mouse antibody (dilution 1:10,000) to assess gel-loading homogeneity.

### Materials

Unless otherwise stated, all compounds were purchased from the Sigma-Aldrich Company Ltd. (St Louis, MO, USA). Clinical grade human recombinant H2 RLX was kindly provided by Prof. Mario Bigazzi (Foundation for the Research on Relaxin in Cardiovascular and Other Diseases, Prosperius Institute, Florence, Italy), The bicinchoninic acid Protein Assay kit and SuperBlock blocking buffer were from Pierce Biotechnology Inc. (Rockford, IL, USA). Antibodies were from Santa Cruz Biotechnology (Santa Cruz, CA, USA). Luminol ECL was from Amersham (Buckinghamshire, United Kingdom).

### Statistical analysis

All values in both the text and figures are expressed as mean ± SEM for n observations. One-way anova with Dunnett's post-test was performed with the GraphPad Prism Software (San Diego, CA, USA) and *P* values below 0.05 were considered significant.

## Results

### Effect of acute rhRLX administration on I/R-induced renal dysfunction

**Figure 1:**
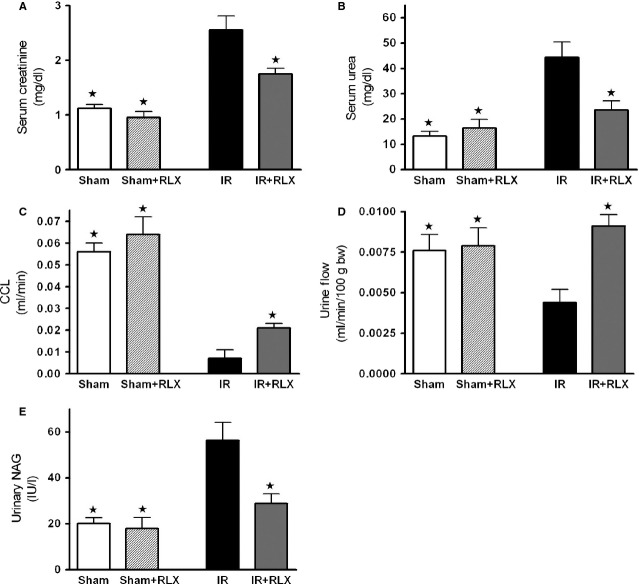
Effect of I/R and rhRLX on renal dysfunction evaluated on blood and urine parameters. Serum creatinine (A), urea (B), creatinine clearance (C), urine flow (D) and urinary N-acetyl-β-glucosaminidase levels (E) were measured after sham operation (Sham) or renal ischaemia–reperfusion injury (IR). Further groups of rats received rhRLX (5 μg/kg, i.v.) at the beginning of reperfusion and again after 3 hrs of reperfusion (Sham+RLX and IR+RLX). Data are expressed as mean ± SEM. ⋆*P* < 0.05 *versus*IR.

Rats that underwent renal I/R exhibited a significant increase in serum levels of urea and creatinine, compared with sham-operated rats (Fig. 1A and B respectively). To discount the possibility of a rapid increase in serum creatinine levels because of increased release of creatinine from muscle during I/R, creatinine clearance was also measured. Ischaemia/reperfusion exposure led to a drastic decrease in creatinine clearance (Fig. 1C) as well as in urine flow (Fig. 1D). Interestingly, administration of rhRLX during reperfusion prevented the increase in the serum concentrations of urea and creatinine and resulted in a significant increase in creatinine clearance and urine flow (Fig. 1A–D), thus indicating improvement in renal injury and glomerular dysfunction. Renal I/R evoked a significant increase in urinary NAG levels, suggesting significant tubular dysfunction, which was markedly reduced by rhRLX administration (Fig. 1E). Conversely, the administration of rhRLX to sham-operated rats had no significant effect on any of the biochemical markers measured.

### Effects of rhRLX on the histological signs of injury caused by I/R

**Figure 2:**
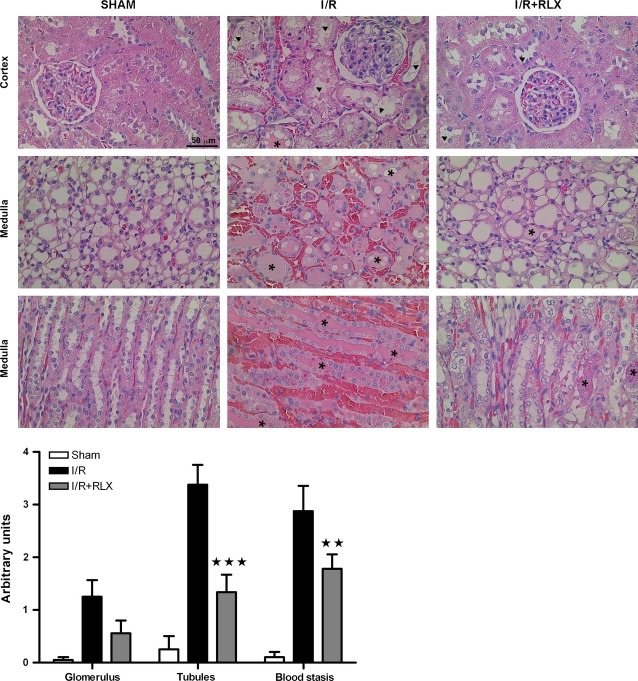
Representative histopathological features of kidney biopsies in the different experimental groups and semi-quantitative assessment of the severity of kidney damage. Upper panels: widespread tubular cell vacuolization, shedding of the tubular epithelial lining (arrowheads) and hyaline tubular casts (asterisks) are seen in the renal cortex and medulla; the interstitial connective tissue shows dilated microvessels filled with blood and sparse haemorrhage foci. Below panel: severity scoring of the histological damage. Significance of differences: ⋆⋆*P* < 0.01 and ⋆⋆⋆*P* < 0.001 *versus*IR.

Figure 2 depicts representative histopathological features of the kidney (cortex and medulla) from rats belonging to the different experimental groups. When compared with the normal kidney morphology of the sham-operated rats, the samples taken from the animals undergoing renal I/R showed typical features of glomerular, tubular and vascular injury. In particular, large tissue areas in both the renal cortex and medulla showed widespread tubular cell vacuolization with reduced or absent ruffled border, accompanied by focal necrosis, shedding of the tubular epithelial lining and formation of hyaline tubular casts. The interstitial connective tissue showed very dilated microvessels filled with blood (peliosis) and sparse haemorrhage foci. Glomeruli in the renal cortex also showed cell microvacuolation and occasional blood extravasation in the Bowman capsule. Of note, rhRLX administration at reperfusion markedly reduced these renal abnormalities, the most evident changes being tubular cell microvacuolation and a moderate degree of microvascular dilation. Semi-quantitative scoring of kidney injury performed on the histological slides confirmed the visual observations and showed that rhRLX significantly attenuates renal cell damage (Fig. 2).

### Effects of rhRLX on oxidative stress induced by renal I/R injury

**Figure 3:**
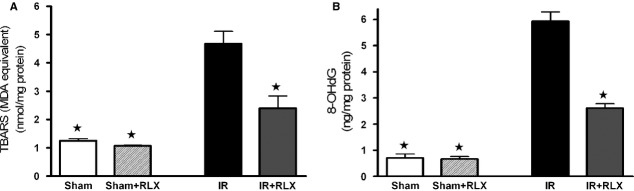
Effects of I/R and rhRLX on lipid peroxidation and free radical-induced DNA in kidney samples. TBARS production (A), 8-OHdG levels (B) were measured subsequent to sham operation (Sham) or renal ischaemia–reperfusion injury (IR) in the absence (vehicle) or presence of rhRLX (5 μg/kg, i.v.; Sham+RLX and IR+RLX). Data are expressed as mean ± SEM. ⋆*P* < 0.05 *versus*IR.

**Figure 4:**
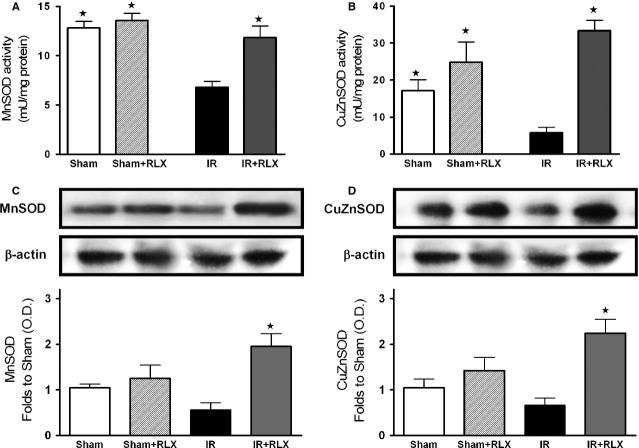
Effects of I/R and rhRLX on activity and expression of manganese-superoxide dismutases (MnSOD) and copper-zinc-superoxide dismutases (CuZnSOD) in kidney samples. Activity (A and B) and protein expression (C and D) of the SOD isoforms mitochondrial MnSOD and cytosolic CuZnSOD were measured in kidney homogenates of sham-operated rats (Sham) and rats that underwent 1-hr ischaemia and 6-hr reperfusion (IR) in the absence or presence of rhRLX (5 μg/kg, i.v.; Sham+RLX and IR+RLX). Each immunoblot is from a single experiment and is representative of three separate experiments. Densitometric analysis of the bands is expressed as relative optical density (O.D.), corrected for the corresponding β-actin contents and normalized using the related sham-operated band. Data are means ± SEM of three separate experiments for Western Blot and five animals/group for SOD activity. ⋆*P* < 0.05 *versus*IR.

Rats that had undergone I/R exhibited a massive increase in tissue markers of oxidative stress, such as TBARS production, an index of peroxidation of cell membrane lipids, and 8-OHdG, a marker of free radical-induced DNA damage (Fig. 3A and B, respectively). The robust increase in TBARS and 8-OHdG levels was blunted by rhRLX administration. Renal I/R injury evoked a significant decrease in the activity of the endogenous antioxidant enzymes MnSOD and CuZnSOD (Fig. 4A and B, respectively), which was associated with a slight suppression of their protein expression (Fig. 4C and D). Interestingly, rhRLX administration almost completely abolished the I/R-induced reduction in MnSOD and CuZnSOD activities and evoked a massive protein up-regulation, above the control levels. On the other hand, rhRLX administration to sham-operated animals had no significant effect on any of the measured markers.

### Effects of rhRLX on inflammatory markers

**Figure 5:**
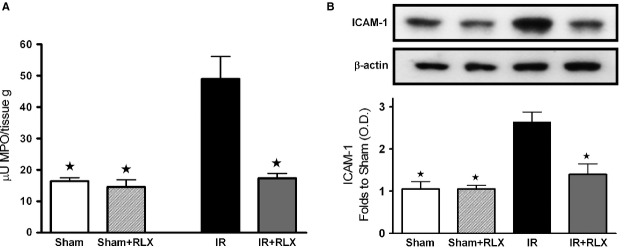
Effects of I/R and rhRLX on leucocyte accumulation in kidney samples. Myeloperoxidase (MPO) activity (A) and ICAM-1 expression (B) were measured in kidney homogenates of sham-operated rats (Sham) and rats that underwent 1-hr ischaemia and 6-hr reperfusion (IR) in the absence or presence of rhRLX (5 μg/kg, i.v.; Sham+RLX and IR+RLX). Each immunoblot is from a single experiment and is representative of three separate experiments. Densitometric analysis of the bands is expressed as relative optical density (O.D.), corrected for the corresponding β-actin contents and normalized using the related sham-operated band. Data are means ± SEM of three separate experiments for Western Blot and five animals/group for MPO. ⋆*P* < 0.05 *versus*IR.

The improvement in the outcome of I/R injury was associated with a marked modulation of the leucocyte accumulation measured in kidney samples at term of reperfusion. As shown in Figure 5A, renal I/R caused a robust increase in MPO activity, a specific leucocyte marker, in comparison with sham-operated rats. In the animals treated with rhRLX during reperfusion, the MPO activity was significantly reduced. The adhesion molecule ICAM-1, which is the endothelial ligand for the neutrophil receptor CD11b/CD18, was barely detectable in the kidney from sham-operated animals, while its expression was strongly induced by I/R (Fig. 5B). Administration of rhRLX drastically reduced the I/R-induced increase in ICAM-1 expression.

**Figure 6:**
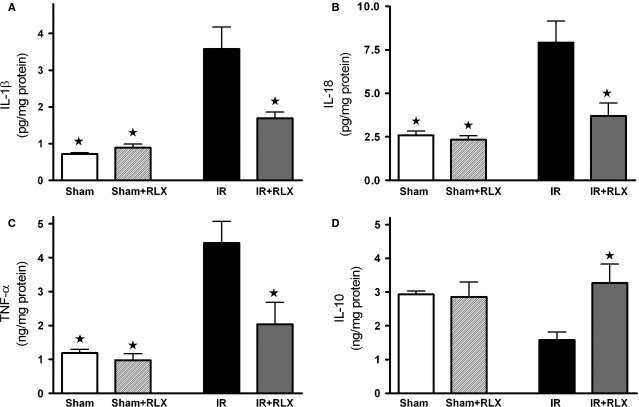
Effects of I/R and rhRLX on cytokine production in kidney samples. IL-1β (A), IL-18 (B), TNF-α (C) and IL-10 (D) levels were measured in the kidney of sham-operated rats (Sham) and rats that underwent 1-hr ischaemia and 6-hr reperfusion (IR) in the absence or presence of rhRLX (5 μg/kg, i.v.; Sham+RLX and IR+RLX). Data are means ± SEM of five animals/group. ⋆*P* < 0.05 *versus*IR.

Interleukin-1β, IL-18 and TNF-α, typical pro-inflammatory cytokines, were significantly increased in renal tissue of ischaemic/reperfused rats, as compared with the sham-operated animals (Fig. 6A–C respectively). Interestingly, administration of rhRLX prevented the I/R-induced rise in IL-1β, IL-18 and TNF-α, levels. When the plasmatic content of the well-known anti-inflammatory cytokine IL-10 was measured (Fig. 6D), a slight decrease in its serum level was detectable in the rats that underwent I/R injury, whereas rhRLX administration reported IL-10 concentration back to values similar to those measured in sham-operated animals.

### Effect of rhRLX on ERK1/2 phosphorylation and iNOS expression in the kidneys of rats that underwent I/R injury

**Figure 7:**
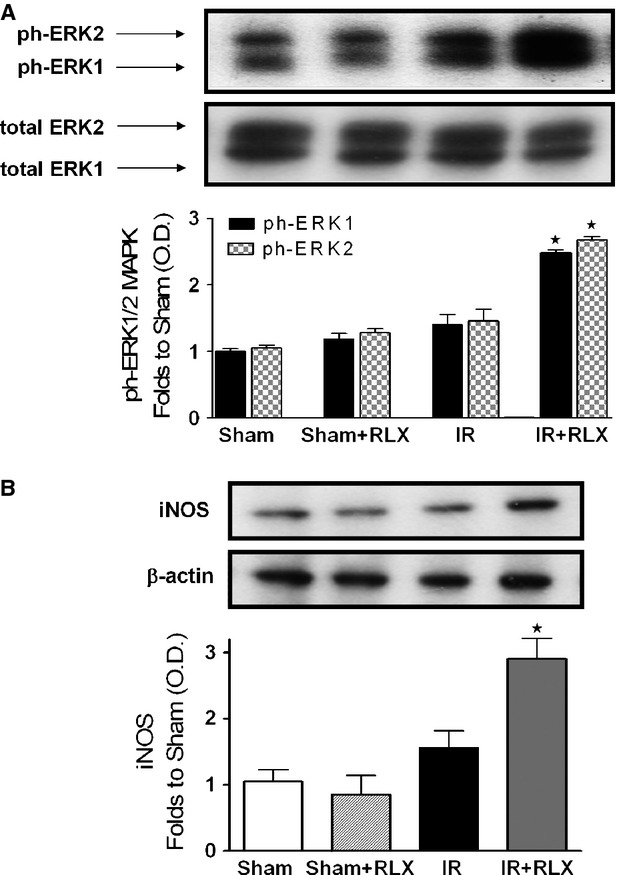
Effects of I/R and rhRLX on ERK phosphorylation and iNOS expression. Representative Western blot and corresponding densitometric analysis of the bands showing phosphorylated (Thr^202^/Tyr^204^) and total ERK1/2 MAPK (A) and iNOS (B) protein expression after 6 hrs of reperfusion in the presence or absence of rhRLX (5 μg/kg, i.v.; Sham+RLX and IR+RLX). Each immunoblot is from a single experiment and is representative of three separate experiments. Densitometric analysis of the related bands is expressed as relative optical density, corrected for the corresponding β-actin contents, and normalized using the related sham-operated band. The data from bands densitometric analysis are means ± SEM of three separate experiments. ⋆*P* < 0.05 *versus*IR.

To gain a better insight into the potential mechanism(s) underlying the observed beneficial effects of rhRLX, we investigated the effects of this hormone on cell signalling pathways known to confer protection to the kidney and that have been previously demonstrated to mediate rhRLX effects in other organs. The phosphorylation of ERK1/2 MAPK was not significantly affected by I/R injury; however, when ischaemic/reperfused rats were treated with rhRLX, we detected a massive increase in ERK phosphorylation (Fig. 7A). As shown in Figure 7B, densitometric analysis of the Western blot bands detected low iNOS protein levels in the kidney obtained from sham-operated animals. I/R injury induced a slight increase in the expression of iNOS, which was maximum in the group of animals treated with rhRLX during reperfusion.

### Effect of rhRLX on the phosphorylation of Akt and eNOS in the kidneys of rats that underwent I/R injury

**Figure 8:**
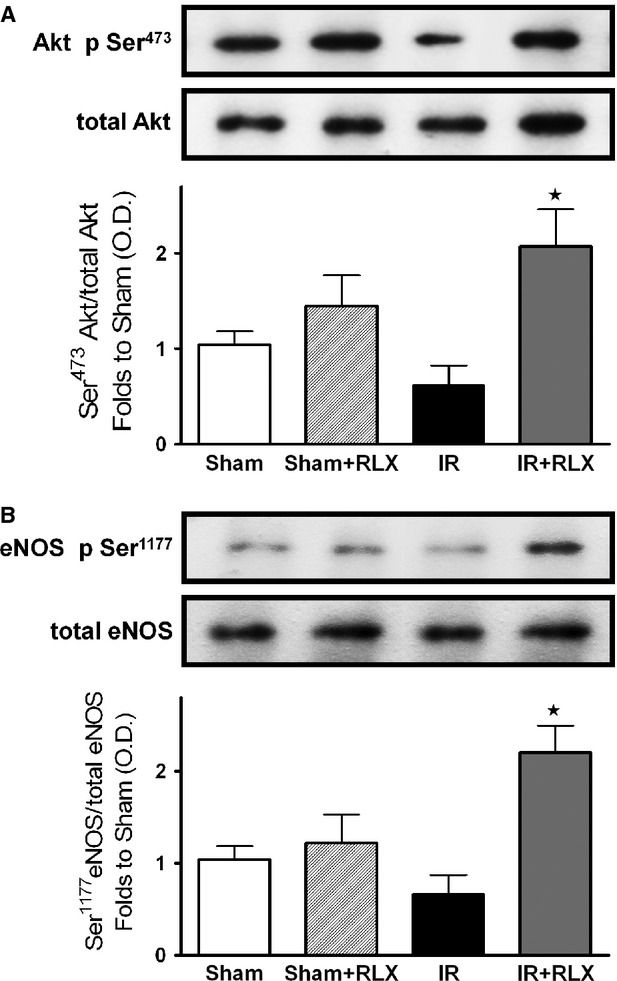
Effects of I/R and rhRLX on Akt and eNOS phosphorylation. Representative Western blot and corresponding densitometric analysis of the bands showing phosphorylated (Ser^473^) and total Akt (A) and phosphorylated (Ser^1177^) and total eNOS in the presence or absence of rhRLX (5 μg/kg, i.v.; Sham+RLX and IR+RLX). Each immunoblot is from a single experiment and is representative of three separate experiments. Densitometric analysis of the related bands is expressed as relative optical density, corrected for the corresponding β-actin contents, and normalized using the related sham-operated band. The data from bands densitometric analysis are means ± SEM of three separate experiments. ⋆*P* < 0.05 *versus*IR.

When compared with sham-operated rats, rats that underwent I/R injury developed significant decreases in the phosphorylation of Akt on Ser473 in reperfused kidney samples (Fig. 8A). Therapeutic administration of rhRLX during reperfusion attenuated the decline in Akt phosphorylation caused by I/R. The degree of the phosphorylation of eNOS on Ser1177 was similar in sham-operated rats and ischaemic/reperfused rats indicating that I/R alone is not sufficient to affect eNOS phosphorylation (Fig. 8B). Treatment of ischaemic/reperfused rats with rhRLX resulted in a twofold increase in the phosphorylation of eNOS when compared with rats exposed to I/R only.

## Discussion

Although recent preclinical and clinical studies have demonstrated that RLX may have important therapeutic potential in chronic kidney diseases, such as renal fibrosis and salt-sensitive hypertension 26, its role in AKI has never been elucidated The present findings offer first experimental evidence that the therapeutic administration of rhRLX at reperfusion significantly reduces renal injury and dysfunction caused by I/R in rats, while it has no effect on normal renal function in sham-operated animals. The dose of rhRLX used in this study has been previously reported to protect other organs against I/R injury and to prevent the development of acute pancreatitis 3,27. Notably, to simulate the clinical conditions, in our experimental model, rhRLX was only applied during reperfusion, indicating that this treatment strategy could be potentially employed in several situations known to result in AKI, including renal transplantation, aortic aneurysm surgery, or X-ray contrast tracer-induced nephropathy, to protect or even rescue a kidney previously challenged by ischaemia. Our results further corroborate and extend the previous findings demonstrating that RLX is capable of ameliorating renal hemodynamics by inducing selective renal vasodilation and hyperfiltration in both rats and humans 29–30. In our experimental model, the improvements in the outcome of renal damage by rhRLX administration were associated with a significant inhibition of both the inflammatory response and oxidative stress induced by I/R. Namely, rhRLX reduced leucocyte adhesion to ischaemic-reperfused vascular endothelium, as suggested by its ability to suppress the expression of the adhesion molecule ICAM-1 and the activity of MPO, selected as typical markers of leucocyte inflammatory recruitment, which were both drastically up-regulated by I/R. At the same time, rhRLX significantly decreased the production of TNF-α, IL-1β and IL-18 in the kidney of animals that underwent I/R injury. Interestingly, this effect was associated with increased level of the anti-inflammatory cytokine IL-10, suggesting that RLX may operate a shift from a pro-inflammatory to an anti-inflammatory status. These results are consistent with previous reports demonstrating the role of RLX as a potent inhibitory factor in early vascular inflammation with prominent inhibitory effects on the expression of cytokines and adhesion molecules 31,32. The attenuated inflammatory response caused by rhRLX treatment may also account for the decrease in tissue markers of oxidative stress, thus supporting the notion that release of ROS from activated leucocytes provides a major contribution to peroxidation of lipid membranes and free radical-induced DNA damage in the kidney. Besides, a direct effect of RLX on oxidative stress has also been recently demonstrated by Dschietzig *et al*. 34, showing that RLX stimulates CuZnSOD expression in rat aortic rings, by increasing the CuZnSOD promoter activity at different time-points. Our findings are in keeping with previous studies from our and other research groups showing that RLX exerts beneficial effects against organ ischaemic damage by reducing local leucocyte recruitment and oxidative stress 3,4. Accordingly, RLX has also been proposed as a protective substance in preservation solutions for lung and liver transplantation 5,35. Despite these intriguing data and the evidence that the kidney is the organ of greatest uptake of exogenously administered RLX 19, the specific signal transduction pathway by which RLX exerts its effects in the kidney remains to be fully elucidated. Previous studies have demonstrated that several renal biological actions of RLX, including its potent antifibrotic effects, are mediated by functional activation of the relaxin receptor RXFP1, which is expressed by specific renal cells, such as mesangial cells and myofibroblasts 37–38. RXFP1 signalling is complex, involving multiple pathways depending on the cell type under investigation; however, recent evidence suggests a key role for the nitric oxide pathway in mediating major renovascular effects of RLX 39. For instance, Sasser *et al*. 40 have demonstrated that RLX was ineffective in preventing chronic renal injury during administration of the nitric oxide synthase inhibitor N(ω)-nitro-l-arginine methyl ester (L-NAME), suggesting that the renoprotective effects of RLX are dependent on a functional NOS system. Although the exact signalling mechanisms of RXFP1 were beyond the scope of this study, we could demonstrate an involvement of the nitric oxide pathway in the RLX-mediated effects reported here: in fact, RLX administration was associated with eNOS activation and induction of iNOS expression, resulting in enhanced formation of nitric oxide in the microcirculation. In conditions associated with I/R, the enhanced formation of nitric oxide is beneficial, as it can cause local vasodilation, inhibit adhesion of platelets and leucocytes and promote angiogenesis 41. There is good evidence that agents that release nitric oxide or enhance the formation of endogenous nitric oxide attenuate organ injury/dysfunction in AKI 42–43. By a nitric oxide-dependent mechanism, RLX has been shown to strongly inhibit neutrophil activation, thereby reducing free radical generation, chemotaxis and platelet aggregation 44–45. Therefore, the reduced oxidative stress status and leucocyte activation here reported may be explained, at least in part, by the ability of RLX to up-regulate the NOS/nitric oxide pathway. Previous studies in cultured human endothelial cells have shown that RLX can evoke eNOS activation by phosphorylation of specific serine residues in Akt 46. Akt is a member of the phosphoinositide 3-kinase signal transduction enzyme family which, upon phosphorylation by its upstream regulator, can modulate inflammatory responses and apoptosis 47. A reduction in the activation of this important survival pathway has been recently demonstrated to make the kidney more susceptible to I/R insult 48–49. Here, we show that RLX caused a robust increase in Akt phosphorylation. This indicates a significant Akt activation, which in turn could promote eNOS phosphorylation and renal protection. An additional contribution to the regulatory effects of RLX on nitric oxide pathway may rely on its ability to affect ERK1/2 MAPK pathway, which is another important signal for cell survival 50. ERK activation protects renal epithelial cells from oxidative injury 51 and, particularly relevant to this study, it leads to iNOS induction in renal epithelial cells 52, renal myofibrobalsts 53, vascular smooth muscle cells 54 and murine macrophages 55. As we documented increased ERK1/2 activation in the presence of RLX, we propose that MAPK activation by RLX is, at least in part, responsible for the RLX-mediated modulation of iNOS expression. However, it must be underlined that ERK1/2 and Akt activation by RLX was recorded at 6 hrs after reperfusion. As RLX has a short serum half-life in rodents 19, we cannot rule out the possibility that RLX evokes an early intracellular signalling cascade leading to late ERK and Akt activation, thus resulting in increased NOS activity/expression.

In conclusion, this study provides first experimental evidence that acute RLX administration during reperfusion attenuates the renal dysfunction and injury caused by I/R in the rat and that these renoprotective effects of RLX involve the activation of eNOS and up-regulation of iNOS, possibly secondary to activation of Akt and ERK1/2, respectively. The modulation of the nitric oxide pathway appears a crucial mechanism through which RLX selectively inhibits the inflammatory response and oxidative stress sparkled by renal I/R. Overall, these findings provide further evidence to the concept that RLX may be regarded as a therapeutic tool in diseases characterized pathogenically by vascular dysfunction and impaired nitric oxide production.
